# Quantification of cell migration: metrics selection to model application

**DOI:** 10.3389/fcell.2023.1155882

**Published:** 2023-05-15

**Authors:** Yang Hu, Matthew L. Becker, Rebecca Kuntz Willits

**Affiliations:** ^1^ Department of Chemical Engineering, College of Engineering, Northeastern University, Boston, MA, United States; ^2^ Departments of Chemistry, Mechanical Engineering and Materials Science, Biomedical Engineering and Orthopedic Surgery, Duke University, Durham, NC, United States; ^3^ Department of Bioengineering, College of Engineering, Northeastern University, Boston, MA, United States

**Keywords:** individual cell migration, diffusion, brownian, anamolous diffusion, cell motility analysis

## Abstract

Cell migration plays an essential role in physiological and pathological states, such as immune response, tissue generation and tumor development. This phenomenon can occur spontaneously or it can be triggered by an external stimuli, including biochemical, mechanical, or electrical cues that induce or direct cells to migrate. The migratory response to these cues is foundational to several fields including neuroscience, cancer and regenerative medicine. Various platforms are available to qualitatively and quantitatively measure cell migration, making the measurements of cell motility straight-forward. Migratory behavior must be analyzed by multiple metrics and then models to connect the measurements to physiological meaning. This review will focus on describing and quantifying cell movement for individual cell migration.

## 1 Introduction

Cell movement is essential throughout the lifespan of an organism. Cells transport passively with blood circulation throughout the body, but also actively migrate through and within tissues. Cell migration is critical to a number of fundamental biological processes, such as stem cell migration during embryogenesis (de Lucas et al., 2018), angiogenesis (Yang et al., 2020), and wound healing (Rodrigues et al., 2019), but is also important to disease states, such as metastasis during tumor development (Wu et al., 2021). Cell migration has long been studied (Angevine and Sidman, 1961; Lauffenburger and Horwitz, 1996; Ridley et al., 2003; du Roure et al., 2005; Peyton and Putnam, 2005; Cattin et al., 2015; Motta et al., 2019; Cavanaugh et al., 2022; Brunetti et al., 2021), with a wide array of studies examining how and why cells move (Isenberg et al., 2009; Roussos et al., 2011; Cortese et al., 2014; Wen et al., 2015), defining exogenous cues that quantitatively impact cell motility. It has long been argued that quantitative characterization of cell migration is critical to permit rigorous comparisons (Dunn and Brown, 1987; Stokes et al., 1991). To improve medical interventions or understand fundamental progression of disease, a quantitative understanding cell migration is critical, including factors that direct or regulate cell movement.

The chosen analytical method must capture subtle differences in cell migration to accurately describe the impact of the conditions on the cell behavior. Cells experiencing individual cell migration have very short duration or no intercellular connections during the entire migration process. *In vivo*, the migration of individual cells can be readily seen with immune cell migration, e.g., neutrophil emigration from the blood stream to a site of infection. To fully explore the relationship between cell migration and quantitative parameters to assess cell migration, this review will focus on characterizing and quantifying cell movement for individual cell migration.

## 2 Analytical metrics for characterization

### 2.1 Cell trajectory

**FIGURE 1:**
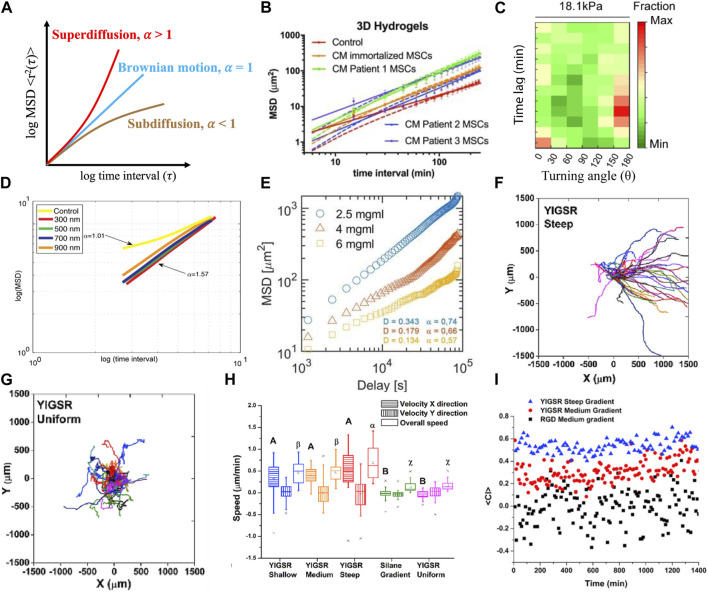
Demonstration of models and metrics commonly used to describe and quantify cell migration. **(A)** Drawing of idealized MSD curves for Brownian motion, subdiffusive and superdiffusive states are defined by fit of the exponent (α) of time interval (τ). See ref (Codling et al., 2008) for further information. **(B)** An anomalous diffusion model (solid line) possessed better fits to describe mesenchymal stem cell migration in 3D hydrogels than a persistent random walk model (dashed line) for all conditions (Luzhansky et al., 2018). **(C)** Immature dendritic cells migrated less persistently with low persistent time and high turning angle distribution under mechanical loaded imposed by 18.1 kPa gels (Choi et al., 2021). **(D)** MSD and superdiffusive migration was used to describe Schwann cell migration on aligned nanofibers of various diameters (Cavanaugh et al., 2022). **(E)** Macrophage migration was subdiffusive in different concentrations of collagen hydrogels (Pérez-Rodríguez et al., 2022). **(F–I)** Individual trajectories of Schwann cells were used to describe migratory response to a peptide concentration gradient, with bias migration on steep peptide concentration profiles **(F)** and random migration was observed on uniform peptide **(G)**; velocities **(H)** and chemotactic index **(I)** of cells were increased as the profile of peptide concentration gradient increased (Motta et al., 2019).

In migration experiments, cell trajectories are tracked via time lapse microscopy, and these paths are then used to calculate a multitude of descriptors described below. Several plug-in applications are available to automatically track cell position over time (Chakrabarti et al., 2018; Fazeli et al., 2020; Hu et al., 2021); however, cells can also be tracked manually by tracing the cell in each image over time and using the center of mass to determine the x, y position in 2D. Tracking in 3D is done similarly using the x, y and z position, or 2D projection of the cell. These positions are then collected over time to provide a basis for the remainder of the analyses presented below. In addition, it is useful to normalize the trajectories to a 0,0 starting position to evaluate the randomness of movement. Motta et al. (Figures 1F, 1. G) described cell trajectory using a visual tool to identity random or directional migration, and showed that medium and steep YIGSR (C**YIGSR** (Cys-Tyr-Ile-Gly-Ser-Arg)) concentration gradients bias Schwann cell (SC) migration while shallow and uniform concentration profiles do not (Motta et al., 2019). The visualization of the cell trajectories is a way to get an overview of how cells are moving and if there are specific motions or bias that may impact the analysis.

### 2.2 Velocity

The raw data generated from cell trajectories are generally positional coordinates in the *x* and *y*-axis, and for 3D data, a *z*-axis. For simplicity, we will focus on 2D migration here. These sets of data can be used to quantify instantaneous and overall average velocities. Instantaneous velocity in *x* and *y* direction of two consecutive positions of an individual cell can be calculated as follows:

$${\vec{v}}_{x,n}=\frac{r_{x,n}\left(t_{i+1}\right)-r_{x,n}\left(t_{i}\right)}{\tau }∙{\vec{e}}_{x}$$ (1)

$${\vec{v}}_{y,n}=\frac{r_{y,n}\left(t_{i+1}\right)-r_{y,n}\left(t_{i}\right)}{\tau }∙{\vec{e}}_{y}$$ (2)

Where τ is the time interval between image captures; n refers to the individual cell and varies from 1 … N, which is the total number of cells tracked; $r_{x,n}\left(t_{i+1}\right)$ and $r_{x,n}\left(t_{i}\right)$ denotes two successive points in *x* direction, where t varies from 0:τ: (f-1)*τ, and f is the total number of frames; $r_{y,n}\left(t_{i+1}\right)$ and $r_{y,n}\left(t_{i}\right)$ denotes two successive points in *y* direction, while $r_{x,n}\left(t_{i+1}\right)$ and $r_{x,n}\left(t_{i}\right)$ denotes two successive points in *x* direction. To describe the average directional velocity, each of the instantaneous velocities are summed and divided by the total number of points, while the overall average instantaneous velocities for a cell population are then calculated based for all of the cells in a population. All velocity terms are vectors and have a magnitude and direction.

In 2D systems, instantaneous velocities can reveal, to a certain extent, the stepwise mode of cell migration, particularly when cells are exposed to external cues in the form of gradients (e.g., chemotaxis, haptotaxis, electrotaxis, or durotaxis). Because the x and y components are isolated, one could detect a bias in migration if the cells primarily moved in one direction. Instantaneous velocities can show detailed response with respect to sampling interval, explaining the transient effect of exogenous cues or self-response to a cell population (Figure 1H) (Maiuri et al., 2015; Motta et al., 2019). It can also show environmental impacts on cell motility (Wu et al., 2014). However, it should be noted that the instantaneous velocity, which can be positive or negative, can be close to zero as a cell moves back and forth. Overall average velocity can be used to describe the global response of a cell population the whole time, which can further to correlate with the effect of external stimuli (Palecek et al., 1997; Luzhansky et al., 2018).

### 2.3 Mean squared displacement (MSD)

MSD is a measurement of displacement of a single cell or group of cells traveling over a particular duration. MSD can be generally classified to time average squared displacement (TASD) and ensemble average square displacement (EASD) (Qian et al., 1991; Chon et al., 1997). TASD is calculated by the following formula within one cell trajectory:

$$\langle r_{n}^{2}\left(\tau \right)\rangle =\langle {\left[r_{x,n}\left(t+\tau \right)-r_{x,n}\left(t\right)\right]}^{2}+{\left[r_{y,n}\left(t+\tau \right)-r_{y,n}\left(t\right)\right]}^{2}\rangle$$ (3)

where *n =* 1,2,3, … represents a single cell migratory path, $\langle r_{n}^{2}\left(\tau \right)\rangle$ denotes the average TASD of a single cell at interval τ. TASD can be calculated over non-overlapping intervals or overlapping intervals. Simply, for overlapping intervals, one would step through the frames including the displacements for every instance of τ; for an interval 2τ, interval 1 could be frame 3 - frame 1 and interval 2 frame 4—frame 2, where interval 1 and interval 2 consider overlapping frames. For non-overlapping intervals, one would skip frames based on τ; for this same interval example, interval 1 would be frame 3-frame 1 and interval 2 would be frame 5-frame 3, with no overlapping of the frames. Since one cell corresponds to one TASD curve, analyzing a large number of cells in one plot can be extremely problematic and unrepresentative, whether graphically or statistically. Therefore, representation of the average mean squared displacement is typical in presentation (Figure 1), and confidence intervals can be added to evaluate differences. Mean squared displacement feeds into most models, and therefore not only can be used to describe migration via plotting log-log with time but can be further evaluated using best fit as described below.

### 2.4 Turning angle distribution

Migration angles are a powerful metric to elucidate the effect of external cues on cells over time. The distribution of these angles, or turning angle distribution (TAD), is used to characterize cell migration behavior and can be classified into two types, global or relative TAD. The global TAD, denoted by θ (-π < θ < π), describes the angle of current direction with respect to a fixed coordinate system (either x or *y*-axis) (Meijering et al., 2011; Yu et al., 2021). The relative TAD, denoted by φ (-π < φ < π), describes the angle relative the previous cell path vector (Mokhtari et al., 2013; Choi et al., 2021; Yu et al., 2021). Global and relative TAD can then be easily back calculated from instantaneous velocities. Global TAD provides an overall view of cell bias while relative TAD provides information about persistence at each position over the time course, allowing researchers to have a clearer and deeper understanding of the cell dynamics between sequential time points. For example, global TAD for mast cells on rigid substrates was independent of the stiffness, describing that the cells had no directional migration.; however, relative TAD (Figure 1D) (Choi et al., 2021) was either 0° or 180°, indicating the cells moved back and forth (Choi et al., 2021; Yu et al., 2021). A polar distribution of TAD can be used to illustrate that cells move along the direction (homodromous or heterdromous) of external cues, while uniform distribution of TAD implies random migration (Masuzzo et al., 2017; Werner et al., 2019; Choi et al., 2021). Therefore, TAD can be analyzed for both global and local assessment of cell migration with typical experimental time lapse capture of cell migration.

### 2.5 Straightness and chemotactic index

Straightness and chemotactic index are measurements of the path of a single cell or a cell population. Straightness index (SI) examines the straightness of cell trajectories, it is often interpreted as a directionality or confinement ratio (Beltman et al., 2009; Gorelik and Gautreau, 2014; Masuzzo et al., 2015). SI is calculated by the ratio of the net displacement of a cell to the total traveled length. Because the experimentally measured total displacement is always less than actual travelled distance in real time, the value of SI can fluctuate between 0 (moving back to the origin) and 1 (perfectly directed cell track). SI can be calculated by following formula:

$$SI=\frac{d_{net}}{d_{total}}$$ (4)

$d_{net}$ represents the displacement between start and end point of a cell path, which is the Euclidean distance. $d_{total}$ represents total traveled length at the time interval between the two points. Between two points adjacent in time, this value is 1; however, as longer time intervals are evaluated, d_net_ becomes smaller relative to total distance traveled.

The chemotactic index (CI) is a another quantitative measurement describing the directionality of cell migration to the direction of a gradients, also called forward migration index (Foxman et al., 1999) or McCutcheon index (McCutcheon, 1946). CI is defined as the distance a cell travels in the direction of chemotactic source divided by the total path length. CI ranges from −1 to +1, with cells migrating either opposed (negative) or in the direction of (positive) the gradient. When CI closes to 0 (Figure 1I) (Motta et al., 2019), no chemotaxis is assumed. CI is calculated by following formula:

$$CI=\frac{d_{directional}}{d_{total}}$$ (5)

where $d_{directional}$ denotes the distance the cell travels in the direction of the gradient, $d_{total}$ means the total path length during the time interval, calculated as in SI. CI plotted with sampling interval can provide a stepwise picture of how cells respond to the surrounding environment.

## 3 Generalized quantitative models for cell migration

### 3.1 Random diffusion model

Models using a persistent random walk to describe cell motility have long been the standard in the field. This random diffusion model is similar to Brownian motion, where each individual cell has equal probability to move in any direction (Uhlenbeck and Ornstein, 1930; Klafter et al., 1996). The Ornstein–Uhlenbeck (OU) process, defined by Langevin equation, has been considered as the prototype of Persistent Random Walk model for individual cell migration (Uhlenbeck and Ornstein, 1930) (Dunn and Brown, 1987). Based on a 1D OU process, in 1942 Doob derived a foundational equation for random motility:

$$MSD=\frac{\alpha }{\beta^{3}}\left(\beta T-1+e^{-\beta T}\right)$$ (6)

where α and β are fitted parameters to the MSD over time (T) (Doob, 1942). The above equation is adjusted to 2 or 3D by multiplying the right-hand side by 2 or 3, respectively through geometric correlations. By fitting the MSD of a cell population, fundamental parameters such as persistence and speed can be calculated (Estabridis et al., 2018; Luzhansky et al., 2018); however *speed* or *persistence* as it relates to α or β must be clearly defined, as authors can express these fit parameters α and β differently (see (Stokes et al., 1991) for further information). This traditional random walk model is ubiquitous to parameterize random cell migration in a 2D environment (see ref (Masoliver et al., 1993) for more details). Additionally, Stokes extended this baseline correlation by accounting for cell migration bias with a chemoattractant (Stokes et al., 1991). However, this model is not as useful within 3D matrices because of discrepancies in fundamental assumptions, such as velocity autocorrelation and Gaussian distribution of velocities (Selmeczi et al., 2005; Kim et al., 2008; Takagi et al., 2008), and therefore the anomalous diffusion model has been more frequently used in these cases.

### 3.2 Anomalous diffusion models

In contrast to random migration models, anomalous diffusion describes the non-Brownian motion of traced particles. This motion can be classified to sub- or super-diffusive by examining a power-law behavior of $MSD\propto \tau^{\alpha }$ , where τ is time interval and α is the anomalous diffusion exponent; 0≤α<1 corresponds to subdiffusive behavior (Figure 1E) and 1<α≤2 corresponds to superdiffusive behavior (Figure 1D). When α = 1, the motion of the particle is Brownian (Figures 1A, D) and the persistent random walk fits well. Various anomalous diffusion models fit a wide range of cell types, both on engineered 2D surface and 3D scaffolds (Metzler and Klafter, 2000; Harris et al., 2012; Huda et al., 2018); see ref (Codling et al., 2008; Vlahos et al., 2008; Chen et al., 2010; Höfling and Franosch, 2013) for more details. However, these non-Brownian models (Dieterich et al., 2008), e.g., Levy walks, are complex to evaluate and have yet to be generalized across cell types or widely used. Generally, these models are used where the persistent random walk model fails - in the subdiffusive regime (Figure 1E) (Wu et al., 2014; Pérez-Rodríguez et al., 2022) - but is also potentially worthwhile to further explore in the superdiffusive regime with non-Gaussian distributions of velocity or position.

## 4 Discussion

Excellent reviews exist to support the choice of migratory models (Carlsson and Sept, 2008; Dieterich et al., 2008; Rangarajan and Zaman, 2008; Metzler et al., 2014). *In vitro* cell migration is often studied with engineered biomaterials or exogenous cues, therefore the most common situations of prioritizing parameter selection are summarized in Table 1. The focus in the discussion below is on the advantages and disadvantages of the parameters.

**TABLE 1:** Priority rankings, strength and limitations of metrics selection for most common scenarios.

	Exogenous cues^a^			Strength	Limitations
	Uniform concentration	Concentration gradient	None cue		
Velocity	^c^	^b^	^c^	Discover transient and overall cell response to exogenous cues; Provide directional insights; Transferable to speed	Highly depend on various independent factors, such as number of cells being analyzed, cell type, status of cells, experimental environment, etc.
TAD	^d^	^c^	^d^	Provide global overview and transient persistence	Highly depend on τ and total duration; Need to be combined with MSD to reveal gradient effect
MSD	^c^	^b^	^c^	Relate cell migration to cues effect in detail by stepwise evaluation	Need to distinguish TASD and EASD carefully because of ergodicity is easy to be ignored
				Discover migration pattern; Can be joint used with migration models	
CI, SI	^c^	^c^	^d^	Useful to discover the effect of gradient profiles; Provide persistence of straightness of migration	Depend on total duration and τ

^a^ Uniform type of cues profile refers to exogeneous cues are at uniform concentration, for example, chemokinesis and haptokinesis, etc. Gradient type of cues profiles refers to exogenous cues are at gradient concentration, for example, chemotaxis and durotaxis, etc. None refers to there is no exogeneous cues, for example, intrinsic cell migration.

^b^ First priority.

^c^ Second priority.

^d^ Third priority.

Velocity is simple to calculate after cell trajectories are tracked. It is an important index to understand underlying mechanisms and systematic impact to cell migration (Gorelik and Gautreau, 2015; Byrne Kate et al., 2016). However, the resulting values are correlated to the sample size, which means the number of tracked cells will have an impact (Kramer et al., 2013; Wu et al., 2014). Cell velocity may be different between species, strain, or sex (Rigaud et al., 2008; Klein and Flanagan, 2016; Eruslanov et al., 2017), reducing the emphasis on the values of velocity and moving toward a focus on relative, statistical evaluation that can better represent population behavior based on Gaussian distribution; a small sample size cannot accurately represent migratory behavior (Schönbrodt and Perugini, 2013; Krithikadatta, 2014). Velocity also depends on the status of cells, for example, the velocity of activated lymphocytes is different than naïve (Chang et al., 1979; Miller et al., 2002; Bhat et al., 2017); macrophages migration velocity is also different in resting and stimulated state (Lee et al., 2020). Physical confinement can also significantly alter cell velocity (Paul et al., 2016; Hui and Pang, 2019).

MSD is an important index that is widely used to quantify cell migration and fitted to diffusion models (Figure 1). One advantage of using MSD is that it can be used with other indices to explore the relationship with environment impact (substrate, scaffold) (Angelini et al., 2010; Isomursu et al., 2022). As noted above, the slope generated from log(MSD)-log (τ) curve, which is the exponent α or the anomalous diffusion exponent, is a powerful tool to describe the cell population in response to various exogeneous cues (Alarcón et al., 2005; Sarris et al., 2012; De la Fuente and López, 2020). One limitation is that most researchers reported MSD results by assuming their system is ergodic–where TASD and EASD are equal. However, TASD and EASD are not interchangeable (Manzo and Garcia-Parajo, 2015) and the differences will be more significant for smaller sample size (Michalet, 2010; Ernst and Köhler, 2013a; Ernst and Kohler, 2013b). Another limitation is that sampling interval can impact MSD (Ernst and Kohler, 2013b; Manzo and Garcia-Parajo, 2015); therefore the time interval of time lapse should be selected within the range of persistent time to be able to fit to a model (Dunn, 1983; Harley et al., 2008; Li et al., 2008; Gorelik and Gautreau, 2014; Luzhansky et al., 2018).

Looking at the TAD can provide long or short duration information about the path during cell migration. One common shortcoming of TAD analysis is that TAD is highly dependent on the sampling interval, τ. Loosley et al. simulated the scenario of long and short capture time intervals for the same single cell migration trajectory (Loosley et al., 2015) and demonstrated that relative TAD tends to be a Gaussian distribution at shorter τ, meaning a large distribution of angles are relatively flat (close to 0°) because fewer time points are recorded at longer τ. When the sampling interval is close to infinity, more detailed angles can be revealed. This results in a TAD curve that is more flattened. Comparing TAD to the CI demonstrated the interval dependency of these parameters (OˈBrien et al., 2014), so it is not clear that both parameters would be valuable.

SI as a measure of cell migration can provide information on the directedness of the paths, in addition to being a parameter that is used in other diffusion-based calculations on the changes from a freely-moving molecule. Limiting its application, the numeric value of SI tends to 0 as the tracking duration increases to infinity (the denominator is significantly greater than the numerator). One way to overcome this situation is calculating the SI within a particular time duration, but it may not reflect the migratory behavior over the entire experimental duration. Another way is to multiply SI by the square root of duration, generating a new version of SI, known as the corrected SI (Beltman et al., 2009). However, the corrected SI is not unitless and not restricted between 0 and 1. Similarly, the experimental sampling gap between two consecutive frames should be shorter than the typical persistence for CI. Because the sampling time may not be known *a priori*, determining the proper timing may necessitate more experiments.

Random cell migration on 2D substrates is well described by persistent random walk model (Tranquillo and Lauffenburger, 1987; Stokes et al., 1991). However, bioengineered 3D scaffolds are capable of incorporating cells to better mimic *in vivo* conditions. Unlike 2D substrates where surrounding physical disturbance are hardly any, cells are obviously subjected to physical interactions when migrating in 3D scaffold (Kim et al., 2008), meaning the movement is often confined, causing the migration of the cell population to be subdiffusive. Researchers have found random walk models cannot well describe subdiffusive cell migration in 3D engineering environments (Kim et al., 2008; Luzhansky et al., 2018). For cells migrating in superdiffusive model, both random walk and anomalous models work equally with minor differences (Luzhansky et al., 2018). Although there is not a one-for-all model, overall, when high portion of subdiffusive cells are observed, the anomalous diffusion model can better overall describe 3D cell migration than a random walk model.

Metrics are ranked based on the presence and type of exogenous cues (Table 1). Cues in gradient form, such as chemotaxis and haptotaxis, can normally bias cell migration, whereas uniform cues will enhance/depress cell migration in all direction. To evaluate cell motility with the presence of exogenous cues, velocity and MSD are two prioritized metrics to consider. These two metrics are powerful to further examine the concentration-dependent or gradient-dependent effect of exogenous cues in a stepwise fashion. SI and CI are useful to examine the directness of cell paths but are less detailed in evaluating multiple uniform profiles. Without exogenous cues, MSD and velocity are still to be able to describe stepwise intrinsic cell response and concentration-dependent effects. However, TAD, SI and CI are expected to be uniformly distributed and close to 0.

Besides choosing appropriate quantification indices to accurately describe cell motility, it is crucial to optimize experimental parameters such as cell seeding density, acquisition rate and duration, size of capture area, intensity and exposure time of objectives, and environmental settings, such as temperature. Assuring the movement of individual cells is a priority, and therefore, cell seeding density needs to be sufficiently high for viability, but low enough so that contact between cells is minimized during the capture. Acquisition rate and capture duration that is too slow will be incapable of capturing the migration pattern; however, faster acquisition or duration can lead to excessive amounts of data. The balance tends to be to capture data slightly faster than the persistence time. The volume of capture can also limit the acquisition rate due to relative speed of the camera/computer. Cell labeling to allow for improved analysis must be carefully considered; the amount of fluorescent dyes (often in mM or nM) used to label cells needs to be sufficient but necessary to avoid phototoxicity (Icha et al., 2017). Similarly, the exposure time needed due to the capture area may further alter labeling and timing. Two other parameters to consider include selecting which cells to track along with the total number of cells to collect. While not all report the exact number of cell tracks embedded in each parameter, approximately 100 cells over at least 3 different experiments provide good population analysis (Kuntz and Saltzman, 1997; Zaman et al., 2006; Jain et al., 2012). Bias and errors in tracking exist whether cells are manually tracked or tracked using automation; additional specifics to avoid biased results are detailed in a review for immune cell migration (Beltman et al., 2009; Svensson et al., 2018), which can be readily extended to other cell types. Images can be computationally expensive, particularly with long duration tracking, and tracking individual paths is time consuming; therefore, where fewer cells are tracked, care must be taken in generalizing the results (Dolde et al., 2021; Cavanaugh et al., 2022). Faster moving cells may require shorter tracking durations than slow moving cells, but ultimately, similar amounts of data are collected (Gorelik and Gautreau, 2014). Clearly, compromises exist when using time lapse microscopy for recording cell migration.

As individual cells migrate, they can migrate in a random or in a directed manner. Consistent and appropriate modeling and calculation of parameters is important to allow for comparative evaluation. With the ease of use of microscopy and improved computational computing power, it is important to evaluate cell paths over time to quantify their migration and include sufficient individual cell paths to provide statistical comparisons. In parameter quantification, migration models can further provide insight on a cell population, but many of the parameters may overlap or not be as useful in the overall evaluation due to the way they are calculated. Therefore, this review provides a metrics-level and model-level descriptions to gain a fundamental understanding of the parameters themselves and their potential use in analyzing a migration experiment.
